# Comparison of vildagliptin and sitagliptin in patients with type 2 diabetes and severe renal impairment: a randomised clinical trial

**DOI:** 10.1007/s00125-015-3655-z

**Published:** 2015-06-12

**Authors:** Wolfgang Kothny, Valentina Lukashevich, James E. Foley, Marc S. Rendell, Anja Schweizer

**Affiliations:** Novartis Pharma AG, Postfach, CH-4002 Basel, Switzerland; Novartis Pharmaceuticals Corporation, East Hanover, NJ USA; Creighton University School of Medicine, Omaha, NE USA; Association of Diabetes Investigators, Omaha, NE USA

**Keywords:** Renal impairment, Sitagliptin, Type 2 diabetes, Vildagliptin

## Abstract

**Aims/hypothesis:**

There are limited data comparing dipeptidyl peptidase-4 (DPP-4) inhibitors directly. We compared the safety and efficacy of vildagliptin and sitagliptin in patients with type 2 diabetes and severe renal impairment (RI).

**Methods:**

This study was a parallel-arm, randomised, multicentre, double-blind, 24 week study conducted in 87 centres across Brazil and the USA. Patients with type 2 diabetes, either drug naive or treated with any glucose-lowering agents, who had inadequate glycaemic control (HbA_1c_ 6.5–10.0% [48–86 mmol/mol]) and an estimated GFR <30 ml min^−1^ [1.73 m]^−2^ were randomised (via interactive voice response technology) to vildagliptin 50 mg once daily or sitagliptin 25 mg once daily. These doses are recommended in this patient population and considered maximally effective. Participants, investigators and the sponsor were blinded to group assignment. Efficacy endpoints included change in HbA_1c_ and fasting plasma glucose (FPG) at all visits and the primary safety endpoint was assessment of treatment-emergent adverse events.

**Results:**

In total, 148 patients were randomised, 83 to vildagliptin and 65 to sitagliptin. All patients were analysed. After 24 weeks, the adjusted mean change in HbA_1c_ was −0.54% (5.9 mmol/mol) from a baseline of 7.52% (59 mmol/mol) with vildagliptin and −0.56% (6.1 mmol/mol) from a baseline of 7.80% (62 mmol/mol) with sitagliptin (*p* = 0.874). FPG decreased by 0.47 ± 0.37 mmol/l with vildagliptin and increased by 0.16 ± 0.43 mmol/l with sitagliptin (*p* = 0.185). Both treatments were well tolerated with overall similar safety profiles.

**Conclusions/interpretation:**

At their recommended doses for severe RI, vildagliptin (50 mg once daily) compared with sitagliptin (25 mg once daily) demonstrated similar efficacy and both drugs were well tolerated. This study provides further support for the use of DPP-4 inhibitors in patients with severe RI.

*Trial registration:* ClinicalTrials.gov NCT00616811 (completed)

*Funding:* This study was planned and conducted by Novartis

**Electronic supplementary material:**

The online version of this article (doi:10.1007/s00125-015-3655-z) contains peer-reviewed but unedited supplementary material, which is available to authorised users.

## Introduction

Renal impairment (RI) is very common in patients with type 2 diabetes [1], as diabetes is the leading cause of kidney failure and end-stage renal disease (ESRD) [2]. In particular, the management of patients with type 2 diabetes and severe RI poses a vast challenge, as therapeutic options are limited because of contraindications and/or increased risk of hypoglycaemia in this patient population [3, 4]. Hypoglycaemia is more common in patients with RI because of decreased renal gluconeogenesis [5], and in particular, overexposure to insulin secretagogues or exogenous insulin is often associated with an increased risk of hypoglycaemia [6].

Dipeptidyl peptidase-4 (DPP-4) inhibitors such as vildagliptin and sitagliptin are generally well tolerated, and are approved for use in patients with severe RI. Owing to their glucose-dependent mechanism of action, they are generally associated with a low risk of hypoglycaemia and are an attractive treatment option for these difficult-to-treat patients [7]. All DPP-4 inhibitors improve glycaemic control by extending the meal-induced increases in glucagon-like peptide-1 (GLP-1) and glucose-dependent insulinotropic polypeptide (GIP) for several hours by slowing the rate of inactivation of these peptides. There are differences in the mechanisms of action of DPP-4 inhibitors, in particular their catalytic binding kinetics [8], which may translate into clinical differences. For example, vildagliptin blocks DPP-4 through substrate-like binding to the active site of the enzyme for an extended time [8]. By contrast, sitagliptin exerts its effect through competitive enzyme inhibition [8, 9]. Only vildagliptin has been shown to block the inactivation of GLP-1 and GIP between meals and overnight [8, 9]. However, there are limited data comparing the various agents directly.

We report here the efficacy and safety/tolerability of vildagliptin compared with sitagliptin in patients with type 2 diabetes and severe RI, with a focus on glycaemic control relative to hypoglycaemic risk.

## Methods

### Study design

This study was a multicentre, randomised, parallel-arm, double-blind, 24 week, clinical trial of vildagliptin (50 mg once daily) and sitagliptin (25 mg once daily) in patients with type 2 diabetes and severe RI (ClinicalTrials.gov registration no. NCT00616811). The primary objective of the study was to evaluate the safety and tolerability of both treatments in this patient population. The study was conducted between January 2008 and October 2010. Participants were recruited as outpatients in 87 centres across Brazil (6) and the USA (81).

Key inclusion criteria for this study included age 18–85 years, BMI 18–42 kg/m^2^, HbA_1c_ 6.5–10.0% (48–86 mmol/mol), type 2 diabetes either untreated (no glucose-lowering medication in the past 8 weeks) or treated with a stable dose of sulfonylurea, thiazolidinedione, meglitinide or insulin, as monotherapy or in combination (for at least 4 weeks), and severe RI (estimated GFR [eGFR] by the Modification of Diet in Renal Disease [MDRD] formula <30 ml min^−1^ [1.73 m]^−2^). Patients were excluded if they had a history of renal transplant, significant cardiovascular history within 6 months, liver disease, abnormal liver function tests (alanine transaminase [ALT] >2× upper limit of normal [ULN], aspartate transaminase >2× ULN or total bilirubin >2× ULN and/or direct bilirubin >ULN) or any treatment that is contraindicated (i.e. metformin) in the severe RI population. The initial protocol excluded patients undergoing any dialysis, but it was subsequently amended to remove this restriction to facilitate recruitment.

Patients continued their initial background treatment throughout the study. After a 2 week, single-blind, placebo run-in period, eligible patients were randomised using interactive voice response technology (IVRS) to receive either vildagliptin (50 mg once daily) or sitagliptin (25 mg once daily) for 24 weeks in addition to continuing their background treatment, if applicable. IVRS assigned a randomisation number to the patient, which was used to link the patient to a treatment arm and to specify unique medication numbers for the first package of study drug to be dispensed to the patient.

This clinical trial targeted enrolling a population of approximately 33% elderly women as a patient population considered more vulnerable. Therefore, patient randomisation was stratified by a combined age and sex factor (≥65 year old women versus others) and background glucose-lowering medication. Randomisation procedures were performed by the investigator or his/her delegate. The study drugs were supplied by Novartis as tablets, and patients were instructed to take one pill a day orally before breakfast. Patients, investigator staff, persons performing the assessments and data analysts remained blinded to the identity of the treatment from the time of randomisation until database lock. Both medications were used at the doses recommended in the label for patients with severe RI. Rescue medication (insulin addition or intensification) could be administered on or after week 4 if fasting plasma glucose (FPG) was >15 mmol/l, after week 8 if FPG >13.3 mmol/l and after week 16 if FPG >12.2 mmol/l.

### Outcomes

HbA_1c_ and FPG were measured at all visits. An analysis of responder rate was also performed to assess the percentage of patients achieving HbA_1c_ ≤6.5% (48 mmol/mol) and <7.0% (53 mmol/mol). HbA_1c_ and routine biochemistry laboratory assessments were performed by a central laboratory (Covance, Indianapolis, IN, USA).

For assessment of safety and tolerability all treatment-emergent adverse events (AEs) were recorded and evaluated by the investigator for severity and possible relationship to study medication. Hypoglycaemia was defined as symptoms suggestive of low blood glucose confirmed by a self-monitored blood glucose measurement <3.1 mmol/l plasma glucose equivalent.

### Statistical analyses

A total of 150 patients (in a 1.5:1 allocation ratio to vildagliptin 50 mg once daily and sitagliptin 25 mg once daily) were planned to be randomised. Assuming an approximate 35% dropout rate (i.e. patients who did not complete the 24 weeks of treatment), 90 patients randomised to the vildagliptin group would provide approximately 58 patients who completed 24 weeks of treatment. A sample size of 58 patients who completed the 24 week study in the vildagliptin treatment group would have 83% power to observe at least one AE with an underlying rate of 3%. For efficacy variables (HbA_1c_ and FPG), the adjusted mean changes from baseline to endpoint (with last observation carried forward) were compared between treatments using an ANCOVA model, with the baseline value as the covariate, and background therapy, pooled centre and treatment as the classification variables. In addition, the time course of HbA_1c_ values and change from baseline by treatment were tabulated and plotted. Efficacy data were censored at the start of rescue medication. The values presented are means ± SE unless otherwise specified. The safety data were summarised descriptively by treatment. Safety analyses were performed on all collected data regardless of rescue medication.

### Ethics and good clinical practice

The study was conducted in accordance with the Helsinki Declaration of 1975, as revised in 2000 and 2008, and the International Conference on Harmonization/Good Clinical Practice guidelines. The study protocol was approved by an independent ethics committee/institutional review board at each site and all patients provided written informed consent.

## Results

### Patient disposition and patient demographic/clinical characteristics

**Fig. 1:**
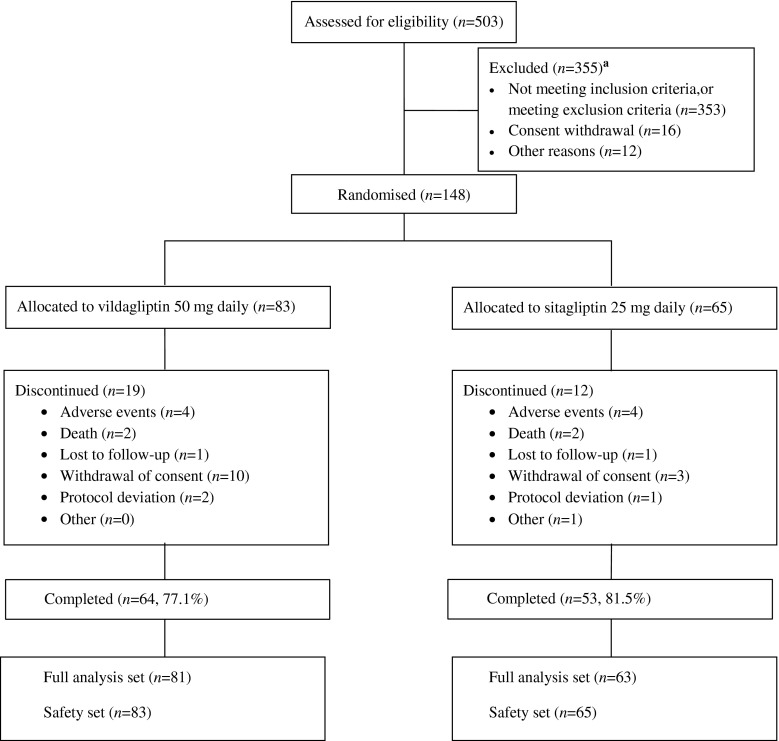
Flow diagram of patient disposition. ^a^More than one reason for discontinuing

A total of 148 patients with type 2 diabetes and severe RI were randomised, 83 patients to vildagliptin (50 mg once daily) and 65 patients to sitagliptin (25 mg once daily), in addition to their stable background glucose-lowering medication. Of the 148 randomised patients, 117 patients completed the study, 64 (77.1%) in the vildagliptin group and 53 (81.5%) in the sitagliptin group, with the most common reasons for discontinuation being withdrawal of consent (vildagliptin 12.0%, sitagliptin 4.6%) and AEs (vildagliptin 4.8%, sitagliptin 6.2%) (Fig. 1). The recruited population with severe RI also included a limited number of patients with ESRD on haemodialysis (six patients in each treatment group).

Table 1 summarises the demographic and clinical characteristics of the patients in the randomised population as well as baseline glucose-lowering medication. There were no clinically meaningful differences between groups in the baseline characteristics. Forty-six (31.1%) patients were elderly women (≥65 years). Mean eGFR (MDRD) was 19.7 ml^−1^ min [1.73 m]^−2^ in the vildagliptin group and 20.4 ml min^−1^ [1.73 m]^−2^ in the sitagliptin group. Patients (48% men/52% women) had a mean age of 66.8 years (with nearly two-thirds ≥65 years), mean BMI of 33.2 kg/m^2^ (with more than two-thirds ≥30 kg/m^2^) and longstanding type 2 diabetes (mean disease duration 19.2 years). Nearly two-thirds of the patients were white, more than 20% were black and about 12% were Hispanic/Latino. Before entering the study, almost all patients (97.3%) were treated with one or more glucose-lowering agents. About 80% of patients received insulin as either monotherapy or combination therapy, at mean doses of 53 U/day in the vildagliptin group and 60 U/day in the sitagliptin group. Mean HbA_1c_ was 7.5% (58 mmol/mol) in the vildagliptin group vs 7.8% (62 mmol/mol) in the sitagliptin group and mean FPG was 8.1 and 7.7 mmol/l, respectively.

**Table 1:** Patient demographics, clinical characteristics and baseline glucose-lowering therapy

Characteristic	Vildagliptin 50 mg once daily (*n* = 83)	Sitagliptin 25 mg once daily (*n* = 65)
eGFR (MDRD) (ml min^−1^ [1.73 m]^−2^)	19.7 ± 6.4	20.4 ± 5.9
Age (years)	66.7 ± 8.8	66.9 ± 9.6
≥65	51 (61.4)	40 (61.5)
Sex		
Male	42 (50.6)	29 (44.6)
Female	41 (49.4)	36 (55.4)
Race		
White	51 (61.4)	40 (61.5)
Black	19 (22.9)	15 (23.1)
Hispanic or Latino	10 (12.0)	7 (10.8)
Other	3 (3.6)	3 (4.6)
BMI (kg/m^2^)	32.7 ± 5.0	33.8 ± 4.8
HbA_1c_ (%)	7.5 ± 0.9	7.8 ± 1.1
HbA_1c_ (mmol/mol)	58 ± 9.8	62 ± 12.0
FPG (mmol/l)	8.1 ± 3.2	7.7 ± 3.0
Duration of type 2 diabetes (years)	18.2 ± 10.4	20.3 ± 10.0
Current glucose-lowering therapy		
None	3 (3.6)	1 (1.5)
Any	80 (96.4)	64 (98.5)
Insulin monotherapy	45 (54.2)	45 (69.2)
Insulin + SUs	11 (13.3)	7 (10.8)
Insulin + TZDs	7 (8.4)	2 (3.1)
SU monotherapy	9 (10.8)	7 (10.8)
Other	8 (9.6)	3 (4.5)

Randomised set

Data are means ± SD or *n* (%)

SU, sulfonylurea; TZD, thiazolidinedione

Patients had concomitant medical conditions expected in patients with type 2 diabetes and severe RI. Hypertension was reported in more than 95%, dyslipidaemia in about 90% and cardiac disorders in nearly 60% of the randomised patients. Nearly all the patients received antihypertensive (95%) and lipid-lowering (88%) medications and more than 60% were taking platelet aggregation inhibitors.

### Glycaemic control and hypoglycaemia

**Fig. 2:**
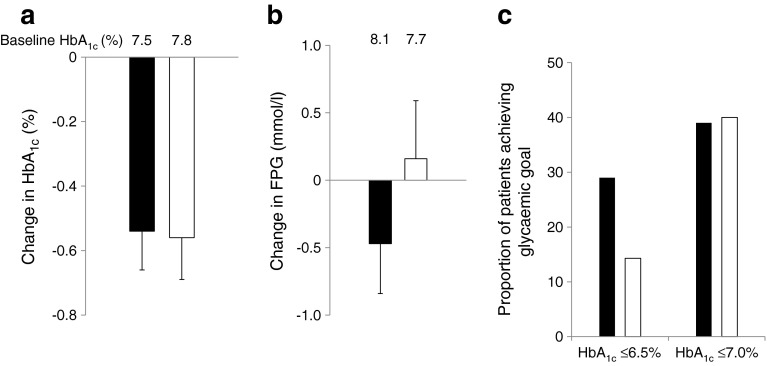
(**a**) Adjusted mean (SE) change in HbA_1c_ from baseline with vildagliptin 50 mg once daily (*n* = 78) or sitagliptin 25 mg once daily (*n* = 62), *p* = 0.874. (**b**) Adjusted mean (SE) change in FPG from baseline with vildagliptin 50 mg once daily (*n* = 79) or sitagliptin 25 mg once daily (*n* = 62), *p* = 0.185. (**c**) Percentage of patients achieving HbA_1c_ ≤6.5% and ≤7.0% with vildagliptin 50 mg once daily (*n* = 69) or sitagliptin 25 mg once daily *n* = 56), *p* = 0.050. Black bars, vildagliptin 50 mg once daily; white bars, sitagliptin 25 mg once daily. To convert values for HbA_1c_ in DCCT % into mmol/mol, subtract 2.15 and multiply by 10.929

The adjusted mean changes in HbA_1c_ and FPG during the 24 week treatment period as well as the percentage of patients achieving a target HbA_1c_ ≤6.5% (48 mmol/mol) are represented in Fig. 2. The adjusted mean change in HbA_1c_ was −0.54% ± 0.12% (5.9 ± 1.3 mmol/mol) from a baseline of 7.52% (59 mmol/mol) in the vildagliptin group and −0.56% ± 0.13% (6.1 ± 1.4 mmol/mol) from a baseline of 7.80% (62 mmol/mol) in the sitagliptin group (*p* = 0.874 for between-group difference; Fig. 2a). A reduction in FPG of 0.47 ± 0.37 mmol/l was seen with vildagliptin, while a slight increase of 0.16 ± 0.43 mmol/l was found with sitagliptin. This difference did not reach statistical significance given the relatively small cohorts (*p* = 0.185 for between-group difference; Fig. 2b). The percentage of patients achieving a target HbA_1c_ ≤7.0% (53 mmol/mol) was similar in both treatment groups (39% vs 40%); however, the proportion of patients achieving a target HbA_1c_ ≤6.5% (48 mmol/mol) in the vildagliptin group was twice that in the sitagliptin group (29.0% vs 14.3%; *p* = 0.050; Fig. 2c). Even though there was a trend towards lower FPG levels in the vildagliptin group, the incidence of hypoglycaemia was similar between the two treatment groups (16% vs 15%). Furthermore, as depicted in Table 2, several AEs probably related to hypoglycaemia were reported less frequently in the vildagliptin group than the sitagliptin group (33% vs 51%). This difference was primarily driven by AEs of hyperhidrosis, tremor and asthaenia, as well as asymptomatic low blood glucose levels.

**Table 2:** Hypoglycaemia and hypoglycaemia-related events

AE	Vildagliptin 50 mg once daily (*n* = 83)	Sitagliptin 25 mg once daily (*n* = 65)
Patients with ≥1 hypoglycaemic event	13 (16)	10 (15)
AEs suggestive of hypoglycaemia	27 (33)	33 (51)
Dizziness	13 (16)	8 (12)
Hyperhidrosis	6 (7)	9 (14)
Tremor	7 (8)	11 (17)
Asthenia	5 (6)	14 (22)
Fatigue	4 (5)	4 (6)
Headache	3 (4)	5 (8)
Hunger	1 (1)	2 (3)
Vision blurred	0	2 (3)
Asymptomatic low blood glucose	4 (5)	6 (9)

Data are *n* (%)

### Overall safety and tolerability

There were no important differences in the overall AE profiles between vildagliptin and sitagliptin. The incidence of AEs (82% vs 86%), serious AEs (24% vs 23%) and discontinuations due to AEs (7% vs 9%) were comparable for vildagliptin and sitagliptin. Deaths were reported in two patients in each group (cardiac arrest and septic shock in the vildagliptin group, and acute pulmonary oedema and asphyxia in the sitagliptin group). Infections and infestations (vildagliptin 35% vs sitagliptin 39%), skin and subcutaneous tissue disorders (25% vs 28%), musculoskeletal and connective tissue disorders (22% vs 23%), cardiac disorders (13% vs 15%), hepatobiliary disorders (0.0% vs 2%) and pancreatitis (0% in both groups) were reported with similar frequencies in both groups. The most commonly reported AE was peripheral oedema, which occurred at a similar frequency in the vildagliptin (23%) and sitagliptin (25%) groups. No deterioration of renal function was observed with either vildagliptin or sitagliptin. Two patients on sitagliptin had ALT elevations (one patient with ALT >3× ULN in the context of a gastritis, one asymptomatic with ALT >5× ULN); both events resolved on treatment. There were no such liver enzyme elevations on vildagliptin. While a limited number of patients with ESRD on haemodialysis were included in the study (*n* = 6 in each group), the safety data did not indicate that these patients receiving vildagliptin or sitagliptin were at an increased risk compared with the overall population with RI.

## Discussion

The study presented here is the first to directly compare efficacy and safety/tolerability of two DPP-4 inhibitors, namely vildagliptin and sitagliptin, in patients with type 2 diabetes and severe RI. The overall HbA_1c_ lowering effect was similar for both drugs and both drugs were well tolerated.

Both drugs in this study were used at their expected maximal effective and recommended doses (in accordance with product labelling) for patients with severe RI. Vildagliptin is mostly hydrolysed to inactive metabolites, with approximately 20% being excreted unchanged [10]. In patients with severe RI, a 50 mg once daily dose of vildagliptin provides full efficacy, as slower elimination effectively doubles the period of time it prevents GLP-1 and GIP inactivation [10, 11]. The HbA_1c_ reductions seen with vildagliptin 50 mg once daily in patients with severe RI were similar to the reductions observed with vildagliptin 50 mg twice daily in patients with preserved renal function [12], and also consistent with HbA_1c_ reductions initially shown in a large, placebo-controlled trial in 515 patients with type 2 diabetes and moderate or severe RI [11]. As sitagliptin is essentially excreted unchanged by the kidney (80% is excreted as the unchanged compound with only a small fraction being metabolised) and peak plasma concentration (*C*_max_) increases approximately fourfold in patients with severe RI, the expected maximal effective dose and the dose recommended on the label in patients with severe RI for sitagliptin is 25 mg once daily [13–16].

The comparative efficacy of 50 mg vildagliptin and 25 mg sitagliptin cannot be determined from this study with certainty as it was not powered to assess relatively small differences in efficacy. In the current study, 80% of patients were on insulin treatment, which likely blunts any differences associated with extending the effects of GLP-1 with vildagliptin during the overnight period. Still, the numerical reduction in FPG with vildagliptin and the slight increase in FPG with sitagliptin are consistent with an effect of vildagliptin during the overnight period. A limitation of the study is that no postprandial blood glucose profiles were obtained. However, studies comparing vildagliptin and sitagliptin in patients with preserved renal function did not find relevant differences in postprandial glucose excursions [17, 18]. Therefore, it is unlikely that this would be the case in our study in patients with RI. The percentage of patients achieving an HbA_1c_ target of ≤6.5% (48 mmol/mol) was higher in the vildagliptin group than the sitagliptin group. This difference is unlikely to reflect a true difference in efficacy between the drugs, and may be a consequence of the slightly lower baseline HbA_1c_ level in the vildagliptin group. Interestingly, this higher responder rate with vildagliptin was not associated with an increased risk of hypoglycaemia with vildagliptin. Overall, the nearly identical drops in HbA_1c_ in our study indicate that the clinical efficacy of both DPP-4 inhibitors is similar in spite of the differences discussed above.

Both drugs were well tolerated. The incidence of hypoglycaemia was similar between the two treatment groups (16% vs 15%). These incidences are low given the vulnerable patient population and that the majority of patients were on insulin treatment. In a study with a similar design, the rates of hypoglycaemia in vildagliptin-treated and placebo-treated patients were of similar magnitude as in our study [11]. The longer extension of the meal-induced increase in GLP-1 and GIP with vildagliptin was not associated with a safety concern in the present study. Of interest, in a prior study in which vildagliptin was dosed at either 50 mg once daily or 50 mg twice daily (i.e. double the recommended dose on the global label) in patients with ESRD, both dosing regimens were well tolerated [19]. Thus, this and other clinical trials demonstrated a good safety and tolerability profile of the entire DPP-4 inhibitor class in populations with severe RI, irrespective of the degree of renal excretion or catalytic binding kinetics [7, 11, 16, 20–24].

In summary, this study demonstrates that vildagliptin 50 mg once daily and sitagliptin 25 mg once daily have similar efficacy and safety profiles in patients with severe RI, supporting the use of DPP-4 inhibitors in patients with severe RI.

## Electronic supplementary material

ESM List of participating investigators (PDF 12 kb)

## Abbreviations

AE: Adverse event
ALT: Alanine transaminase
DPP-4: Dipeptidyl peptidase-4
eGFR: Estimated GFR
ESRD: End-stage renal disease
FPG: Fasting plasma glucose
GIP: Glucose-dependent insulinotropic polypeptide
GLP-1: Glucagon-like peptide-1
IVRS: Interactive voice response technology
MDRD: Modification of diet in renal disease
RI: Renal impairment
ULN: Upper limit of normal
