# Supplementary material for: Comparison of vildagliptin and sitagliptin in patients with type 2 diabetes and severe renal impairment: a randomised clinical trial
Source: Diabetologia. 2015 Jun 12;58(9):2020–6. doi: 10.1007/s00125-015-3655-z (PMC4526592; doi:10.1007/s00125-015-3655-z)
Supplement: Supplementary file 1 — (PDF 12 kb) [file 125_2015_3655_MOESM1_ESM.pdf]

Appendix 1

List of participating investigators

Abdul Abdellatif, Bayer College of Medicine, Houston, USA; Ahmad Haidar, Mississippi Medical Research, LLC, Picayune USA; Ahmed Awad, Clinical Research Consultants, LLC, Kansas City, USA; Alan Rauba, Jefferson City Medical Group, Jefferson City, USA; Alan Wynne, Cotton-O'Neil Diabetes & Endocrinology Center, Topeka, USA; Andrea Phillips, Phillips Medical Services, PLLC, Jackson MS, USA; Anil Agarwal, The Ohio State University Medical Center, Columbus, USA; Anjay Rastogi, UCLA Medical Center, Los Angeles, USA; Anna Chang, John Muir Clinical Research, Concord CA, USA; Anuj Bhargava, Iowa Diabetes & Endocrinology Research Center PLC, Des Moines, USA; Arnold Silva, Boise Kidney & Hypertension Institute Meridian, USA; Axel Pflueger, Mayo Clinic Rochester Rochester MN, USA; Bauer Vaughters, Aiken Center for Clinical Research Aiken, USA; Bradley Dixon, University of Iowa, Iowa City, USA; Bruce Samuels, Bruce Samuels LLC, Covington, USA; Carol Wysham, Washington State University at Spokane, Spokane, USA; Daniel Whittington, Dolby Research, LLC, Baton Rouge, USA; David Robertson, Atlanta Diabetes Associates, Atlanta, USA; Deise Carvalho, Hospital Geral do Bonsucesso, Rio de Janeiro, Brazil; Dinorah Nutis, Texas Tech University Health Sciences Center, El Paso, USA; Domenic Sica, Medical College of Virginia, Richmond, USA; Douglas S. Denham, Diabetes & Glandular Diesease Research Associates, P.A., San Antonio, USA; Eduardo Martin, South Florida Nephrology Associates Fort Lauderdale, USA; Elizete Keitel, Complexo Hospitalar Santa Casa de Porto Alegre, Porto Alegre, Brazil; Fernando Almeida, Hospital Santa Lucinda Sorocaba, Brazil; Fernando Trespalacios, Nephrology Associates of South Miami Aventura, USA; Gentii Filho, Hospital de Clínicas da UNICAMP Campinas, Brazil; Geoffrey Block, Denver Nephrology PC Denver, USA; George Fadda, California Institute of Renal Research La Mesa, USA; Gerald Stephanz, Central Utah Clinic American Fork, USA; German Hernandez, German Ramirez, Tampa Bay Nephrology Associates Tampa, USA; Gopal Chemiti, Meritcare Medical Group Fargo, USA; Harmeet Singh, Western Nephrology & Metabolic Bone Disease, Arvada, USA; Jakkidi Reddy, Sierra Clinical Research,

Roseville, USA; James Fidelholtz, Hightop Medical Research Center Cincinnati, USA; James Thrasher, Medical Investigations, Inc. Little Rock, USA; Janet McGill, Washington U School of Medicine, St. Louis, USA; John Gilbert, Clinical Trials of St. Jude Heritage Medical Group, Fullerton, USA; Jonathan Wise, Crescent City Clinical Research Center, Metairie, USA; Joseph Lee, Apex Research of Riverside, Riverside, USA; Juan Velez, Medical University of South Carolina, Charleston, USA; Julio Rosenstock, Dallas Diabetes & Endocrine Center, Dallas, USA; Kadir Mansur, Jacksonville Center for Clinical Research, Jacksonville, USA; Kashif Latif, AM Diabetes and Endocrinology Center, Bartlett, USA; Kashif Munir, Joslin Diabetes Center at North Arundel Hospital, Glen Burnie, USA; Larry Levinson, Larry Levinson, D.O., PA Hollywood, USA; M. Cecilia Lansang, Shands Medical Plaza, Gainesville, USA; Marc Rendell, Creighton Diabetes Center, Omaha, USA; Mario Belledonne, Biolab Research, LLC, Rockville, USA; Markus Wettstein, Hartford Hospital, Hartford, USA; Marwan Kaskas, Northwest Louisiana Nephrology Research, Shreveport, USA; Mary Ann Banerji, SUNY Downstate Medical Center Brooklyn, NY, USA; Matthew Esson, Western Nephrology & Metabolic Bone Disease PC, Westminster, USA; Maysa Cendoroglo, Universidade Federal de São Paulo, São Paulo SP, Brazil; Michael Cox, Cotton-O'Neil Diabetes & Endocrinology Center, Topeka, USA; Michelle Krause, University of Arkanasas for Medical Sciences, Little Rock, USA; Mohamed El-Shahawy, Academic Medical Research Institute, Los Angeles, USA; Moustafa Moustafa, SC Nephrology and Hyptertension Center, Inc, Orangeburg, USA; Norman Fishman, Diabetes and Endocrinology specialist, Inc Chesterfield, USA; Pablo Pergola, Renal Associates, P.A. San Antonio, USA; Patricia Bononi, Renal Endocrine Associates, P. C., Pittsburgh, USA; Patricia Kapsner, University of New Mexico Health Science Center, Albuquerque, USA; Patricia P. Buchanan, Willamette Valley Clinical Studies, Eugene, USA; Paul Underwood, Sonoran Health Specialists, Scottsdale, USA; Peter Bressler, North Texas Endocrine Center, Dallas, USA; Pusadee Suchinda, Sumter Medical Specialists, Sumter, USA; Rajib Bhattacharya, Cray Diabetes Education Center, Kansas City, USA; Ramakant Mulay, Medical Nephrology Associates, Dyersburg, USA; Raul Rodelas, AKDHC Medical Research Services, LLC, Peoria, USA; Renuka Sothinathan, Clinical Research and Consulting Center, Fairfax, USA; Richard Arakaki, University of

Hawaii, Honolulu, USA; Richard Cherlin, Los Gatos, USA; Richard Hranac, Platte Valley Medical Group Kearney, USA; Richard Kelly, Anasazi Internal Medicine Phoenix, USA; Richard Lund, Creighton Nephrology, Omaha, USA; Rodica Pop-Busui, University of Michigan Clinical Trials Office, Ann Arbor, USA; Ruth Weinstock, SUNY - Upstate Medical University Syracuse, NY, USA; Sam Lerman, Center for Diabetes & Endocrine Care Hollywood, USA; Samuel Blumenthal, Zablocki VA Medical Center, Milwaukee, USA; Sérgio Draibe, Universidade Federal de São Paulo, São Paulo, Brazil; Shashi K. Kant, University of Cincinnati Department of Nephrology, Cincinatti, USA; Sherwyn L. Schwartz, Diabetes & Glandular Diesease Research Associates, P.A. San Antonio, USA; Sohail Khan, Florida Medical Clinic, PA, Zephyrhills, USA; Syed Hussain, Theodore Herman, HRRG Orchard Park, NY, USA; Uma Rangaraj, Arthritis and Diabetes Clinic Monroe, USA; Veronica Piziak, Scott & White Hospital, Temple, USA; Vin Tangpricha, Emory Clinic Atlanta, USA; Wei Feng, South Pasadena, USA, and William Zigrang, Burlingame, USA.
